# Relevance of circulating Semaphorin 4A for rheumatoid arthritis response to treatment

**DOI:** 10.1038/s41598-023-41943-3

**Published:** 2023-09-05

**Authors:** Jérôme Avouac, Eloïse Vandebeuque, Alice Combier, Lucile Poiroux, Alexia Steelandt, Margaux Boisson, Virginie Gonzalez, Anne Cauvet, Thomas Barnetche, Marie-Elise Truchetet, Christophe Richez, Yannick Allanore

**Affiliations:** 1 INSERM U1016 and CNRS UMR8104, Institut Cochin, Paris, France; 2 Université Paris Cité, Paris, France; 3 Service de Rhumatologie, Hôpital Cochin, AP-HP. Centre - Université Paris Cité, Université de Paris, 27 Rue du Faubourg Saint-Jacques, 75014 Paris, France; 4 Service de Rhumatologie, FHU ACRONIM, Hôpital Universitaire de Bordeaux, Bordeaux, France

**Keywords:** Predictive markers, Rheumatoid arthritis

## Abstract

The lack of validated tools to predict rheumatoid arthritis (RA) disease course warrants the development of new reliable biomarkers. Our aim was to evaluate the merit of circulating SEMA4A for the prediction of outcomes in patients with RA. In a first cohort of 101 consecutive RA patients followed up for 41 ± 15 months, increased baseline SEMA4A levels were identified as an independent predictor of treatment failure (hazard ratio, HR 2.71, 95% CI 1.14–6.43), defined by the occurrence of patient-reported flares and initiation or change of targeted therapy. The highest predictive value of treatment failure was obtained with the combination of increased circulating SEMA4A and/or Disease Activity Score (DAS) 28-CRP > 3.2 and/or active synovitis on doppler ultrasound (HR 10.42, 95% CI 1.41–76.94). In a second independent cohort of 40 consecutive RA patients who initiated new therapy because of insufficient disease control, baseline SEMA4A levels were significantly higher in patients who further experienced none or moderate response, and SEMA4A concentrations were markedly decreased in the group of patients with good clinical response as compared to non-responders. Circulating SEMA4A appears as an appealing biomarker in RA with ability to predict treatment failure, and with association with response to therapy.

## Introduction

The main objective in rheumatoid arthritis (RA) is to control joint inflammation to prevent irreversible structural bone and cartilage damages^1^. To this end, treatment target is to reach and maintain stringent long-term remission^2^. Treatment algorithms encompass measuring disease activity with composite indices applying a treat-to-target strategy, and use of conventional, biological, and targeted synthetic disease-modifying anti-rheumatic drugs (DMARDs)^3^. However, despite the recent development of several targeted therapies, many issues persist regarding the treatment and the management of RA patients, such as the identification of RA patients requiring tighter disease control through thanks to a more aggressive therapeutic strategy. The lack of validated tools to predict RA disease course and response to treatment warrants the development and validation of new reliable biomarkers that would personalize monitoring and therapeutic options according to the risk of treatment failure^4^. Indeed, nonspecific biomarkers, such as erythrocyte sedimentation rates (ESR) and C-reactive protein (CRP), are partial measures of RA disease activity, often lacking noteworthy sensitivity and specificity. Indeed, ESR and CRP may show normal results despite ongoing disease activity^5^. Moreover, although the prognostic value of anti-citrullinated protein antibodies (ACPA) has been demonstrated, their value in assessing RA disease activity remains unproven.

Recent promising results from our lab support that Semaphorin 4A (SEMA4A) may be a relevant biomarker to monitor the risk of persistent disease activity and treatment response. Increased SEMA4A concentrations have been detected in the serum, synovial fluid, and synovial tissue of patients with RA^6^. In addition, we previously showed that SEMA4A serum levels correlated with multiple clinical, biological, and ultrasound markers of disease activity, including the Disease Activity Score (DAS)-28 and DAS28-CRP composite scores^7^. Following these promising results, our aims herein were to (i) evaluate the merit of circulating SEMA4A to predict RA treatment failure and (ii) study its association with response to therapy.

## Results

### Analysis of cohort 1 from Cochin Hospital, Paris

#### Study population

A total of 101 patients (85 females, 84%) with established RA were included between May 2016 and February 2018. These patients had a mean age of 58 ± 13 years, a mean disease duration of 14 ± 11 years, and a mean follow-up of 41 ± 15 months. Positive rheumatoid factors and anti-CCP antibodies were detected in 80 (79%) and 83 (82%) patients, respectively. Erosions were present in 63 (62%) patients; 70 patients (69%) received corticosteroids (including 9 with a dose > 10 mg/day), 78 received conventional synthetic disease-modifying anti-rheumatic drugs (csDMARDs), including 61 (60%) with MTX, and 59 (58%) received targeted biologic DMARDs (bDMARDs). During the inclusion visit, 13 patients initiated a first line of bDMARD or switched to another bDMARD because of insufficient disease control. Detailed characteristics of our study sample are provided in Table 1.

**Table 1:** Characteristics of patients from the 2 cohorts at baseline.

	Cohort 1 from Paris (n = 101)	Cohort 2 from Bordeaux (n = 40)
Demographics		
Age (years), mean ± SD	58 ± 13	57 ± 14
Females, n (%)	85 (84)	29 (73)
Active smokers, n (%)	23 (23)	ND
Disease characteristics		
Disease duration (years), mean ± SD	14 ± 11	5 ± 6
Positive rheumatoid factor, n (%)	80 (79)	27/34 (79)
Positive anti-CCP2 antibodies, n (%)	83 (82)	28/34 (82)
Erosions on hand/foot X-rays, n (%)	63 (62)	16 (40)
Disease activity		
DAS28, mean ± SD	3.18 ± 1.36	5.12 ± 1.40
DAS28 ≥ 3.2, n (%)	43	34 (85)
ESR (mmH1), mean ± SD	19 ± 17	43 ± 31
ESR > 28 mmH1, n (%)	22 (22)	24 (60)
CRP (mg/L), mean ± SD	8.5 ± 24	16 ± 21
CRP > 10 mg/L, n (%)	20 (20)	15/37 (41)
Function		
HAQ, mean ± SD	1.02 ± 0.85	ND
Treatment received		
Current corticosteroid use, n (%)	70 (69)	26 (65)
Current corticosteroid use, > 10 mg/day, n (%)	9 (9)	0 (0)
Current conventional DMARD use, n (%)	76 (75)	20 (50)
Current MTX use, n (%)	61 (60)	18 (45)
Current targeted biologic therapies, n (%)	59 (58)	10 (25)
Current anti-TNF-α use, n (%)	18 18	ND
Current rituximab use, n (%)	23 (23)	ND
Current tocilizumab use, n (%)	10 (10)	ND
Current abatacept use, n (%)	8 (8)	ND
Initiation of/switch to a new targeted biologic therapy, n (%)	13 (13)	25 (63)

*DAS* disease activity score, *CRP* C-reactive protein, *ESR* erythrocyte sedimentation rate, *HAQ* health assessment questionnaire, *MTX* methotrexate, *TNF* tumor necrosis factor, *ND* no data.

#### Outcomes

The number of annual consecutive visits ranged from 2 to 5 (88 patients with 3 visits, 72 with 4 visits and 65 with 5 visits). Disease flares occurred in 38 patients during the mean follow-up period of 41 ± 15 months. Among these 38 patients, targeted therapy was added or modified in 26 patients because of insufficient disease control: 10 initiated a bDMARD or a targeted synthetic (ts)-DMARD and 16 switched from a bDMARD to a new b- or tsDMARD (Table S1). The mean time to treatment modification was 35 ± 13 months.

#### Primary endpoint: evaluation of the predictive value of SEMA4A for the occurrence of treatment failure

**Figure 1:**
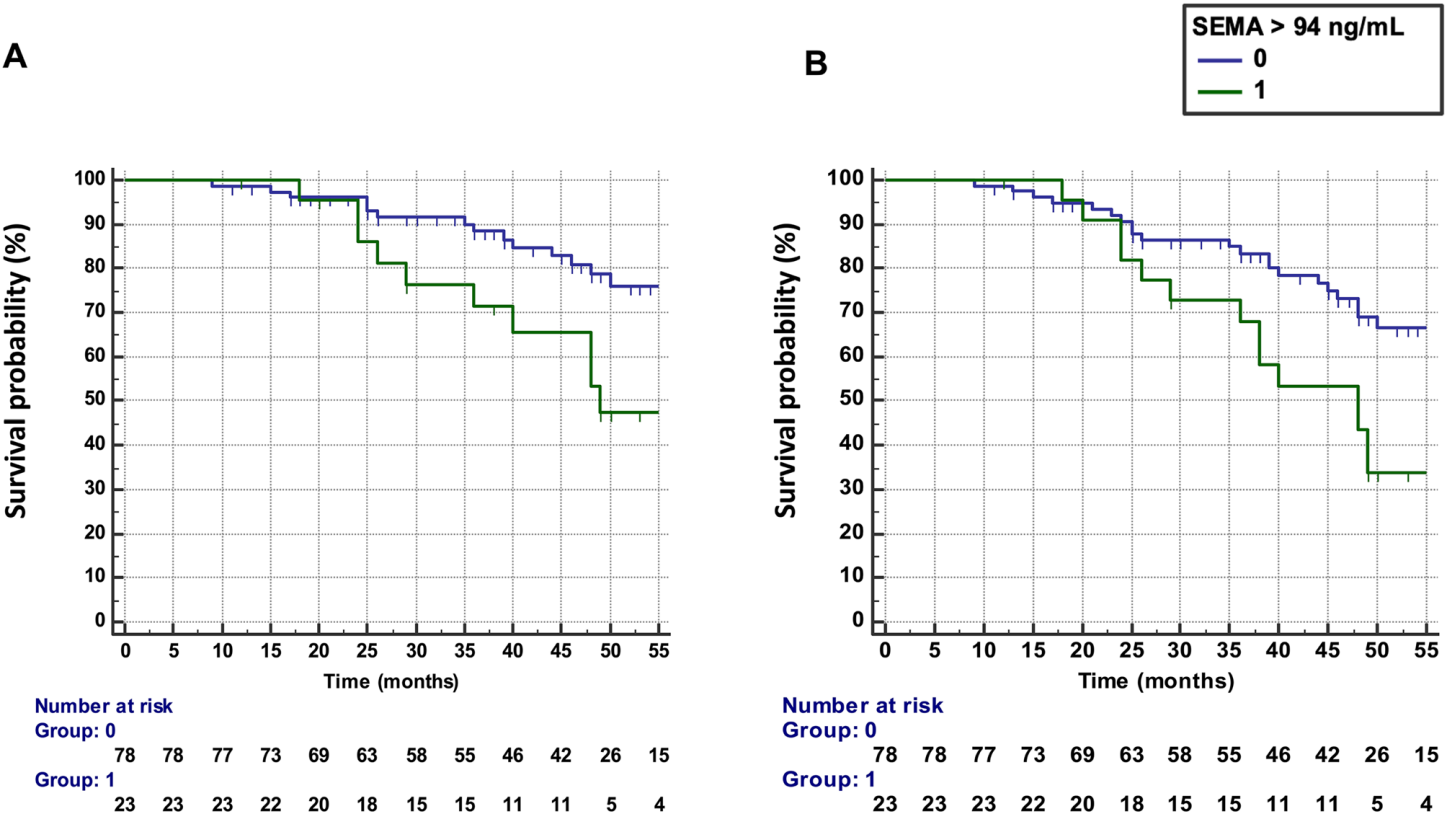
Predictive value of SEMA4A for the progression of rheumatoid arthritis in cohort 1 from Paris. (**A**) Time to treatment failure (defined as flares AND treatment escalation) according to circulating SEMA4A concentrations (≤ or > 94 ng/mL). (**B**) Disease flare-free survival according to circulating SEMA4A concentrations (≤ or > 94 ng/mL).

Baseline SEMA4A levels > 94 ng/mL were predictive of treatment failure, defined by the occurrence of flares AND treatment escalation (n = 26 patients), with an HR of 2.73 (95% CI 1.24–5.96) (Fig. 1A). The results were unchanged after the exclusion of the 13 patients with active disease at baseline requesting the addition or the change of a bDMARD (HR: 2.83, 95% CI 1.14–7.52).

#### Secondary endpoints

Increased SEMA4A levels (> 94 ng/mL) at baseline was predictive of the occurrence of flares (n = 34 patients) during the follow-up period (Fig. 1B) with a hazard ratio (HR) of 2.43 (95% confidence interval, CI 1.27–4.68). The results were unchanged after the exclusion of the 13 patients with active disease at baseline (HR 2.36, 95% CI 1.15–4.89).

**Figure 2:**
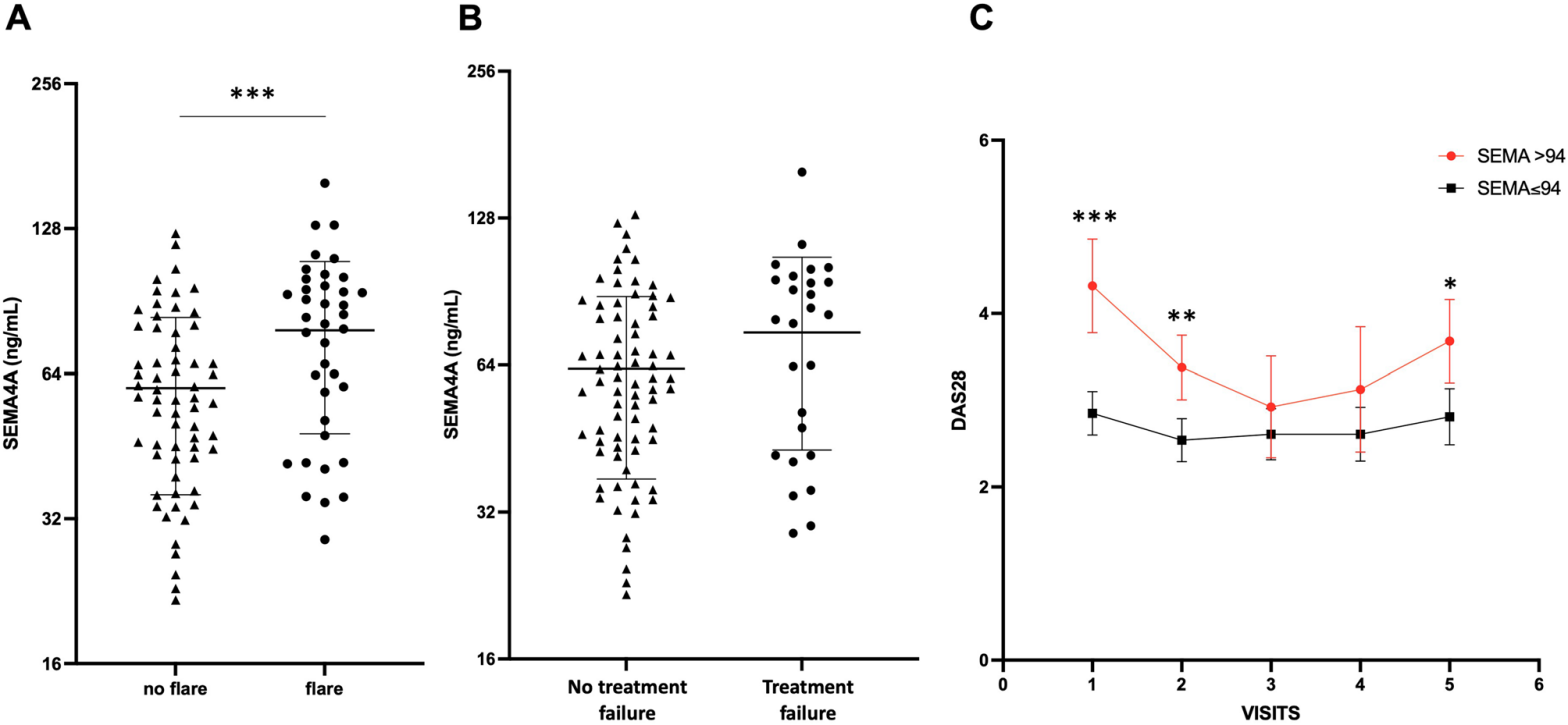
Baseline circulating SEMA4A levels according to the occurrence of (**A**) disease flare or (**B**) treatment failure (defined as flares AND treatment escalation) during the prospective follow-up period in cohort 1 from Paris. (**C**) Course of the DAS28 during the follow-up period according to baseline SEMA4A concentrations (≤ or > 94 ng/mL). All data are shown as the mean ± SEM. *p < 0.05, **p < 0.01 and ***p < 0.001, determined by Student’s t test.

Baseline SEMA4A concentrations were significantly increased in patients experiencing flares during the follow-up period (78 ± 30 ng/mL vs. 60 ± 24 ng/mL, p < 0.001) (Fig. 2A). SEMA4A levels were also significantly higher in the 13 patients with active disease at baseline requesting the addition or the change of a bDMARD compared to the 88 patients with stable treatment (84 ± 33 ng/mL vs. 63 ± 26, p = 0.011). However, although baseline SEMA4A concentrations were higher in patients who experienced flares AND treatment escalation compared to those with stable treatment (75 ± 31 ng/mL vs. 63 ± 26 ng/mL, p = 0.060), this did not reach significance (Fig. 2B). Patients with increased baseline SEMA4A levels maintained higher DAS28 values during the whole follow-up period, with significant differences at visits 1, 2 and 5 (Fig. 2C).

#### Integration of SEMA4A with other predictors of treatment failure

A baseline DAS28 > 3.2 (HR 2.17, 95% CI 1.01–4.72) and the presence of active synovitis, defined by at least a grade 2 doppler activity^8^, detected on at least one joint on power doppler ultrasound (PDUS) (HR 3.60, 95% CI 1.07–12.15) were predictive of the further occurrence of treatment failure. These results were not modified after the exclusion of the 13 patients with active disease at baseline.

Baseline age, disease duration, ACPA or RF positivity, smoking status, presence of erosions, line of targeted DMARDs, treatment with corticosteroids and CRP levels were not predictive of treatment failure (Table 2). Multivariate Cox analyses adjusting for these covariates confirmed that SEMA4A was the single independent predictors of treatment failure (HR 2.71, 95% CI 1.14–6.43).

**Table 2:** Univariate and multivariate Cox analyses to identify independent predictors of treatment failure (primary endpoint) and RA flares (secondary endpoint) in cohort 1 from Paris.

Variable at baseline	Treatment failure: RA flares AND treatment escalation		RA flares	
	Univariate analysis (HR, 95% CI)	Multivariate analysis (HR, 95% CI)	Univariate analysis (HR, 95% CI)	Multivariate analysis (HR, 95% CI)
SEMA4A > 94 ng/mL	2.73 (1.24–5.96)	2.71 (1.14–6.43)	2.43 (1.27–4.68)	2.12 (1.04–4.32)
DAS28 > 3.2	2.17 (1.01–4.72)	2.36 (0.95–5.37)	2.28 (1.20–4.33)	2.57 (1.19–5.56)
Active synovitis on PDUS	3.60 (1.07–12.15)	3.23 (0.90–11.59)	2.80 (1.08–7.29)	2.91 (1.05–8.07)
Age	0.99 (0.96–1.02)	0.99 (0.97–1.03)	0.97 (0.95–1.00)	0.98 (0.96–1.00)
Active smokers	1.23 (0.26–3.89)	1.10 (0.75–1.05)	1.41 (0.35–4.02)	1.29 (0.39–5.09)
Disease duration	1.00 (0.97–1.04)	0.99 (0.96–1.05)	0.99 (0.97–1.02)	1.01 (0.97–1.05)
Positive rheumatoid factor	1.92 (0.58–6.43)	1.22 (0.46–3.60)	2.91 (0.89–9.49)	2.70 (0.88–10.07)
Positive anti-CCP2 antibodies	1.08 (0.37–3.15)	0.97 (0.88–1.12)	1.68 (0.59–4.75)	1.20 (0.39–3.71)
Erosions on hand/foot X-rays	1.85 (0.74–4.62)	2.00 (0.69–5.80)	0.96 (0.50–1.86)	0.97 (0.43–2.16)
CRP levels > 10 mg/L	1.27 (0.50–3.16)	0.94 (0.34–2.59)	1.49 (0.72–3.08)	1.14 (0.52–2.49)
Current treatment with corticosteroids	1.07 (0.42–2.67)	0.80 (0.30–2.16)	0.89 (0.42–1.84)	0.85 (0.39–1.86)
Line of targeted therapy (> 1 line vs. first line)	1.77 (0.48–5.26)	1.63 (0.39–5.98)	1.98 (0.56–6.12)	2.03 (0.41–2.65)

*RA* rheumatoid arthritis, *CRP* C-reactive protein, *HR* hazard ratio, *CI* confidence interval, *PDUS* power doppler ultrasound.

SEM4A was also confirmed as an independent predictor of flares, together with the DAS28 and synovial hyperhemia (Table 2).

**Figure 3:**
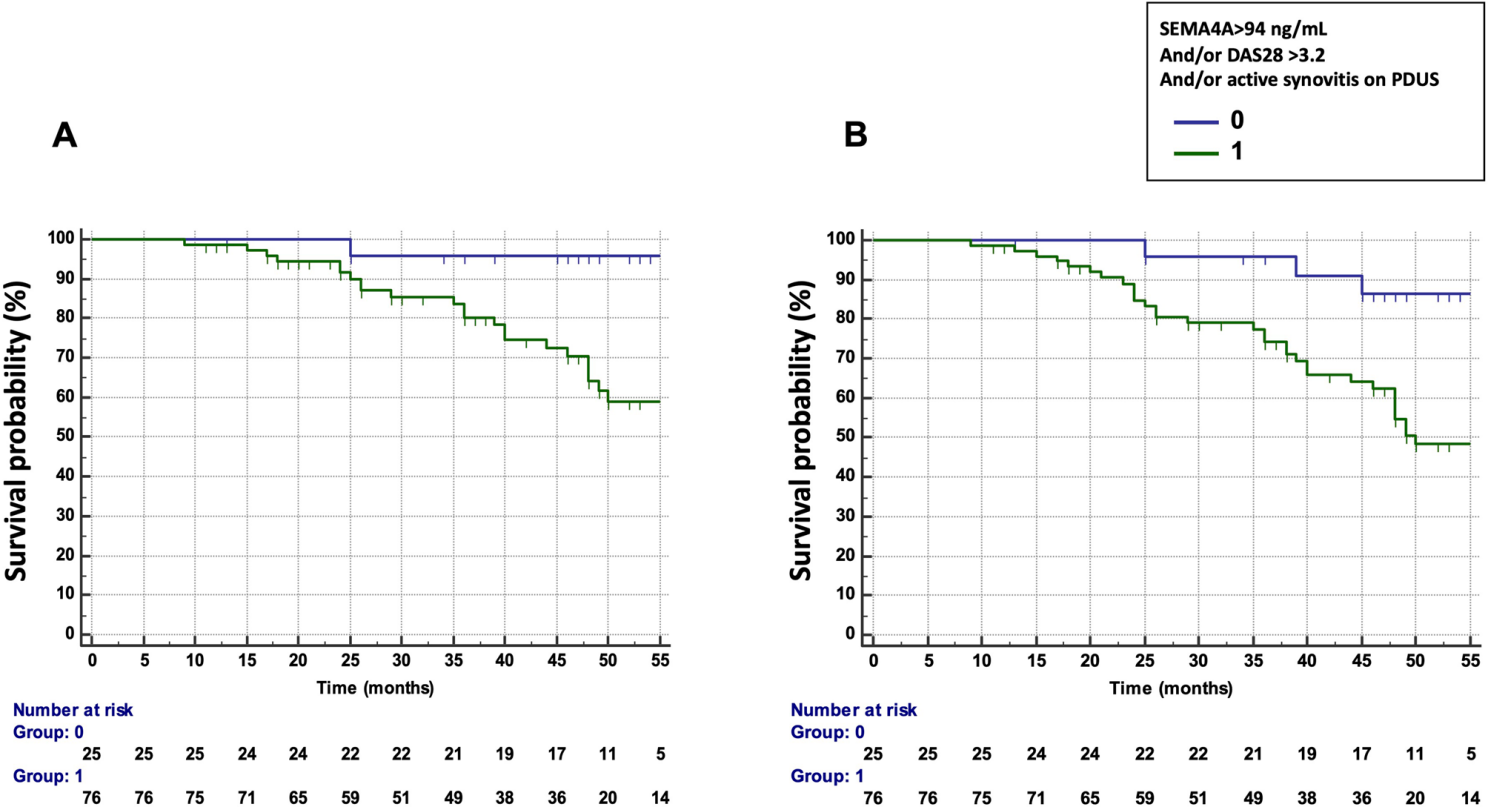
Predictive value of SEMA4A, alone or in combination with a Disease Activity Score (DAS) 28 > 3.2 and/or the presence of active synovitis on power doppler ultrasound (PDUS) in cohort 1 from Paris. (**A**) Time to treatment failure (defined as flares AND treatment escalation) according to circulating SEMA4A concentrations (> 94 ng/mL) and/or a DAS28 > 3.2 and/or the presence of active synovitis on PDUS. (**B**) Disease flare-free survival according to circulating SEMA4A concentrations (> 94 ng/mL) and/or a DAS28 > 3.2 and/or the presence of active synovitis on PDUS.

We next assessed the potential combination of the DAS28, PDUS and SEMA4A concentrations to predict the occurrence of treatment failure and flares (Table 3). The combination which provided the best predictive value was a DAS28 > 3.2 and/or presence of active synovitis on PDUS and/or SEMA4A concentrations > 94 ng/mL (HR 10.42, 95% CI 1.41–76.94 for treatment failure and 4.88, 95% CI 1.50–15.89 for flares) (Fig. 3A,B). Matrix models also highlighted the ability of the combination of these 3 parameters to predict the occurrence of treatment failure and flares (Fig. S1): Treatment failure and RA flare occurred in 53% and 73% of patients with baseline DAS28 > 3.2 and presence of active synovitis on PDUS and SEMA4A concentrations > 94 ng/mL, respectively. Moreover, only a single patient with a DAS28 ≤ 3.2, no active synovitis and SEMA4A ≤ 94 ng/mL experienced treatment failure and RA flares.

**Table 3:** Predictive value of circulating SEMA4A alone or in combination DAS28-CRP and/or active synovitis on power doppler ultrasound for the occurrence of treatment failure (primary endpoint) and RA flares (secondary endpoint) in cohort 1 from Paris.

Variables	Number of patients	Treatment failure: RA flares AND Treatment escalation		Flares	
		Hazard ratio	Hazard ratio	Hazard ratio	95% CI
SEMA4A > 94 ng/mL	23	2.73	1.24–5.96	2.43	1.27–4.68
SEMA4A > 94 ng/mL and/or DAS28 > 3.2	50	2.46	1.10–5.53	2.80	1.41–5.57
SEMA4A > 94 ng/mL and/or active synovitis on PDUS	68	3.60	1.13–10.50	2.47	1.13–5.41
DAS28-CRP > 3.2 and/or active synovitis on PDUS	73	5.69	1.34–24.08	3.13	1.22–8.02
SEMA4A > 94 ng/mL and/or DAS28 > 3.2 and/or active synovitis on PDUS	76	10.42	1.41–76.94	4.88	1.50–15.89

*DAS* disease activity score, *PDUS* power doppler ultrasound, *CI* confidence interval.

#### Predictive value of SEMA4A in the subset of patients with low disease activity or remission

In the 58 patients with DAS28 < 3.2 at baseline, treatment failure occurred in 11 (19%) patients during the observation period. In this population, increased SEMA4A concentration was the single variable that predicted the occurrence of treatment failure (HR 3.50, 95% CI 1.02–12.01). The presence of active synovitis detected on at least one joint on PDUS and other clinical or biological variables did not predict treatment failure (Table S2).

In the 37 patients with a DAS28 < 2.6, treatment failure occurred in 4 patients (11%) and increased SEMA 4A had a trend for predicting treatment failure (HR 3.30, 95% CI 0.82–152.11, p = 0.069).

Increased SEMA4A concentration was also identified as the single predictor of flares (n = 16, 28%) in this subset of 58 patients with DAS28 < 3.2 (HR 3.68, 95% CI 1.33–10.17).

### Analysis of the cohort 2 from Pelegrin Hospital, Bordeaux

#### Study population

A total of 40 patients (29 females, 73%) were included. These patients had a mean age of 57 ± 14 years, a mean disease duration of 5 ± 6 years and active disease with a mean DAS28 of 5.12 ± 1.40. Positive rheumatoid factors and anti-CCP antibodies were detected in 27 (79%) and 28 (82%) patients, respectively. Erosions were present in 16 (40%) patients; 26 patients (65%) received corticosteroids. At the inclusion visit, 15 patients initiated MTX as a first line therapy and 25 started tocilizumab. Tocilizumab initiators were older, had higher disease duration and disease activity, and were more likely to receive corticosteroids than MTX initiators. Detailed characteristics of our study sample are provided in Tables 1 and S3.

#### Analysis of the course of SEMA4A serum levels according to treatment response

Among the 40 included patients, 4 experienced no treatment response, 10 had a moderate response and 26 a good response. As previously observed, baseline SEMA4A levels correlated with the DAS28 (r = 0.29, p = 0.038) and a trend was observed with CRP (r = 0.26, p = 0.10). At month 3, SEMA4A concentrations correlated with the DAS28 and CRP (r = 0.31, p = 0.029 and r = 0.38, p = 0.017, respectively). Moreover, baseline SEMA4A concentrations were significantly increased in active patients at inclusion, defined by a DAS28 > 3.2 (Fig. S2A). Interestingly, baseline SEMA4A levels were significantly higher in patients who further experienced none or moderate response (198 ± 30 ng/mL) compared to patients with good response (176 ± 24 ng/mL, p = 0.035) (Fig. S2B). SEMA4A serum levels were found markedly decreased between m0 and m3 especially in the group of patients with good clinical response (Fig. S2C). This result was observed in the subsets of patients initiating either MTX or tocilizumab (Fig. S2D,E).

## Discussion

SEMA4A has been recently implicated in the pathogenesis of RA through the modulation of immunity and angiogenesis^6, 7, 9^. Preliminary data have suggested that SEMA4A concentrations may be of interest in RA. Indeed, increased SEMA4A serum concentrations were detected in RA patients and were associated with markers of disease activity in 2 independent French cohorts^7^. The herein study brings a further step of validation in 2 prospective cohorts. In the first cohort from Paris, increased baseline SEMA4A levels were able to predict treatment failure in daily practice. In addition, baseline SEMA4A levels identified more active and difficult to treat patients with higher mean DAS28 values during the follow-up period in the first cohort. In the second cohort from Bordeaux, SEMA4A concentrations correlated with disease activity and a good clinical response after 3 months of therapy with MTX or tocilizumab was associated with a marked reduction of SEMA4A serum levels. Although no other study has reported yet the link between SEMA4A and response to therapy in inflammatory diseases, accumulating evidence indicates that semaphorins may be considered as relevant for the assessment of responsiveness to therapy in cancer^10^.

These results replicate and extend the findings obtained in a previous independent prospective cohort of 70 RA patients in remission or low disease activity from the rheumatology department of Montpellier. Circulating SEMA4A concentrations, measured with the same method, were found significantly higher at baseline in the 14 patients who did not maintain sustained remission during the 12-month follow-up, compared to patients who maintained sustained remission^7^. Taken together, these results support that circulating SEMA4A is a relevant candidate biomarker in RA.

SEMA4A was complementary to the DAS28 > 3.2 and the presence of active synovitis on PDUS to predict treatment failure, given the high predictive value of these 3 variables alone or in combination. Circulating SEMA4A may constitute for the clinician an additional red flag indicating the need of a tighter follow-up and potential therapeutic adaptation.

Another important information provided by this study is that patients with baseline DAS28 > 3.2 and/or persistent active synovitis, considered stable by their clinician given the absence of treatment modification during the visit, were more at risk of future disease flare. This support the concept of not keeping a disease active, even if that activity is considered not clinically relevant by the clinician. However, it should be noted that this concept is made challenging by the recruitment in tertiary centers of severe RA patients, with difficult to treat disease, refractory to several treatment lines.

Patient-reported flares with tender and swollen joints were considered as an endpoint in our study since they are associated with worse disease activity and quality of life, as well as radiographic progression^11^. Self-reported flares are substantiated by higher disease activity measures, independently associated with pain and swollen joints, and related to treatment escalation^12^. RA flares have mainly been investigated in patients in remission/low disease activity or in patients tapering drugs. Several potential biomarkers have shown interest in this population, including increased circulating calprotectin, which was detected in RA patients that relapsed within 12 months of tapering anti-rheumatic drugs^13^. Calprotectin serum levels were also associated with the loss of clinical remission and treatment change in RA patients throughout 5 years of follow-up^14^. The multi-biomarker disease activity (MBDA) score has also been identified as a predictor of disease relapse in patients with RA stopping TNF inhibitor treatment^15, 16^. In our study, increased SEMA4A concentrations were also predictive of treatment failure in the subset of patients in remission or low disease activity at baseline, but this finding was extended to our whole patient population, independently of their inflammatory status at inclusion.

Our study included in the first cohort patients with advanced disease who were carefully assessed and phenotyped in a tertiary center with a long-lasting experience in evaluation and care of RA. The study design was prospective and treatment decisions were non-protocolized. Nevertheless, treatment decisions were made in a tertiary referral center with multidisciplinary approach to ensure consensus and uniformity.

Limitations of our studies were the absence of analysis of the CDAI and SDAI which are barely used in clinical practice in our center. In the second cohort, the assessment of response to treatment was assessed only at 3 months and was limited to patients initiating MTX or tocilizumab, without consideration of other class of targeted DMARDs. These results need to be validated at longer term and other therapies should be investigated.

In summary, our study shows that SEMA4A is a a potential biomarker of interest in RA that deserves further validation regarding its comparison with clinical activity indices.

## Methods

### Study design

A prospective observational routine care study was conducted in the Rheumatology departments of Cochin Hospital, Paris and Pelegrin Hospital, Bordeaux. Two independent prospective series of patients were investigated.

All methods were carried out in accordance with relevant guidelines and regulations. The protocol and the informed consent document have received Institutional Review Board/Independent Ethics Committee (IRB/IEC) approval before initiation of the study (Paris: “Comité de Protection des Personnes” Paris Ile de France I, n° CPPIDF-DAP13; Bordeaux: “Comité de Protection des Personnes”, reference DC2014/21). All patients agreed to participate in this study after written informed consent, which was recorded in the medical source file.

### Cohort 1 from Cochin Hospital, Paris

#### Patient samples

We considered our set of 130 patients with available SEMA4A serum concentrations at baseline, whose characteristics have previously been published^7^. Among these patients, 101 had at least one annual follow-up visit and were further included in this study.

#### Setting

Patients were recruited between May 2016 and February 2018. Patients were systematically evaluated in day hospitalization on an annual basis until the end of the study period (August 2021). We only considered patients with at least two consecutive annual visits.

#### Data collection

The detailed methods for the collection of clinical and biological data, as well as ultrasonography assessment have been previously reported^7^. RA disease activity was assessed at each visit by the Disease Activity Score based on the evaluation of 28 joints (DAS28)^17^.

#### SEMA4A concentrations

Serum concentrations of SEMA4A have been previously measured in this cohort^7^. Increased SEMA4A concentrations were defined as values > 94 ng/mL since this cut-off value previously identified RA patients with an “inflammatory profile” with an area under curve of 0.80 (P < 0.001)^7^.

#### Outcomes

We considered at each study visit the occurrence of at least one patient-reported flares with swollen and tender joints since the last visit^12^ and treatment escalation, defined by the initiation or change by the treating physician of the ongoing targeted biologic / synthetic therapy because of insufficient efficacy.

The primary endpoint was the merit of increased SEMA4A concentrations to predict treatment failure defined by the occurrence of flare AND treatment escalation. The secondary endpoint was the value of increased SEMA4A concentrations to predict the occurrence of flares. Other analyses included the comparison of baseline SEMA4A concentrations in patients experiencing or not flares or treatment escalation, and the course of the DAS28 according to baseline SEMA4A concentrations during the follow-up period.

### Cohort 2 from Pelegrin hospital, Bordeaux

#### Patient samples

We analyzed the serum of 40 consecutive RA patients with moderate to severe RA fulfilling the 2010 ACR/EULAR criteria, coming from the TOCIHELPER trial (Characterization of the Effect of Tocilizumab in Vivo and in Vitro on T Follicular Helper Cells in Rheumatoid Arthritis Patients and Consequence on B Cells Maturation, NCT02569736). These patients initiated methotrexate (MTX) or tocilizumab because of insufficient disease control and had available serum and disease activity assessment at inclusion and 3 months after treatment introduction.

#### Setting and data collection

Patients were assessed at treatment initiation and after 3 months. Patient history, physical examination data, current medications, and results of laboratory tests (autoantibody status, erythrocyte sedimentation rate, ESR, elevated if > 28 mm hour-1, CRP concentration, elevated if > 10 mg/L) were collected through the review of medical files. RA disease activity was evaluated by the DAS28. The presence of erosions was assessed by systematic hand and foot x-rays.

#### SEMA4A concentrations

Serum concentrations of SEMA4A have been measured by quantitative ELISAs (SEMA4A Coud-Clone Corp, Katy, TX), in the same location and conditions as the first cohort, as previously described^7^.

#### Outcomes

We analyzed the course of SEMA4A levels according to treatment response. A good response was defined when the delta DAS28 between baseline and 3 months (Δ DAS28) was > 1.2. A moderate response was defined by a Δ DAS28 between 0.6 and 1.2 and the absence of response by a Δ DAS28 < 0.6.

### Statistical analysis

All data are presented as mean values ± standard deviation (SD) or number and percentage (%) for continuous and categorical variables, accordingly. Statistical analysis was performed using GraphPad Prism (v9.1.2) and Medcalc (v18.9.1). We used the unpaired t-test for two-group comparisons (continuous variables) and the chi-square test for differences in frequency (binary variables). In the cohort from Paris, treatment failure or RA flare free survival according to SEMA4A concentrations was estimated by Kaplan–Meier survival curves. To identify predictive factors of treatment failure or RA flares, we used cox proportional-hazard regression. We also constructed risk matrix to assess the predictive values of circulating SEMA4A levels, DAS28-CRP, the presence of active synovitis by PDUS for the occurrence of treatment failure or RA flares. In the cohort from Bordeaux, SEMA 4A concentrations were compared between month 3 and the inclusion visit using the Wilcoxon matched-pairs signed rank test.

### Ethics approval and consent to participate

All patients recruited in the Rheumatology department of Cochin Hospital, Paris, signed a consent form approved by the local institutional review boards (Comité de Protection des Personnes, Paris Ile de France 3). All patients recruited in Bordeaux Hospital signed a consent form approved by the local institutional review boards (Comité de Protection des Personnes, reference DC2014/21).

### Supplementary Information

Supplementary Information.

## Data Availability

All data generated or analysed during this study are included in this published article [and its supplementary information files].

## References

[1] Smolen JS, Aletaha D, Barton A, Burmester GR, Emery P, Firestein GS (2018). Rheumatoid arthritis. Nat. Rev. Dis. Primers..

[2] Solomon DH, Bitton A, Katz JN, Radner H, Brown EM, Fraenkel L (2014). Review: Treat to target in rheumatoid arthritis: Fact, fiction, or hypothesis?. Arthritis Rheumatol..

[3] Smolen JS, Breedveld FC, Burmester GR, Bykerk V, Dougados M, Emery P (2016). Treating rheumatoid arthritis to target: 2014 update of the recommendations of an international task force. Ann. Rheum. Dis..

[4] Shapiro SC (2021). Biomarkers in rheumatoid arthritis. Cureus..

[5] Kay J, Morgacheva O, Messing SP, Kremer JM, Greenberg JD, Reed GW (2014). Clinical disease activity and acute phase reactant levels are discordant among patients with active rheumatoid arthritis: Acute phase reactant levels contribute separately to predicting outcome at one year. Arthritis Res. Ther..

[6] Wang L, Song G, Zheng Y, Tan W, Pan J, Zhao Y (2015). Expression of Semaphorin 4A and its potential role in rheumatoid arthritis. Arthritis Res. Ther..

[7] Avouac J, Pezet S, Vandebeuque E, Orvain C, Gonzalez V, Marin G (2021). Semaphorins: From angiogenesis to inflammation in rheumatoid arthritis. Arthritis Rheumatol..

[8] D'Agostino MA, Terslev L, Aegerter P, Backhaus M, Balint P, Bruyn GA (2017). Scoring ultrasound synovitis in rheumatoid arthritis: a EULAR-OMERACT ultrasound taskforce-Part 1: Definition and development of a standardised, consensus-based scoring system. RMD Open.

[9] Carvalheiro T, Rafael-Vidal C, Malvar-Fernandez B, Lopes AP, Pego-Reigosa JM, Radstake T (2020). Semaphorin4A-Plexin D1 axis induces Th2 and Th17 while represses Th1 skewing in an autocrine manner. Int. J. Mol. Sci..

[10] Mastrantonio R, You H, Tamagnone L (2021). Semaphorins as emerging clinical biomarkers and therapeutic targets in cancer. Theranostics..

[11] Bechman K, Tweehuysen L, Garrood T, Scott DL, Cope AP, Galloway JB (2018). Flares in rheumatoid arthritis patients with low disease activity: Predictability and association with worse clinical outcomes. J. Rheumatol..

[12] Kuettel D, Primdahl J, Weber U, Terslev L, Ostergaard M, Petersen R (2020). Pain and self-reported swollen joints are main drivers of patient-reported flares in rheumatoid arthritis: Results from a 12-month observational study. J. Rheumatol..

[13] de Moel EC, Rech J, Mahler M, Roth J, Vogl T, Schouffoer A (2019). Circulating calprotectin (S100A8/A9) is higher in rheumatoid arthritis patients that relapse within 12 months of tapering anti-rheumatic drugs. Arthritis Res. Ther..

[14] Ramirez J, Cuervo A, Celis R, Ruiz-Esquide V, Castellanos-Moreira R, Narvaez JA (2021). Biomarkers for treatment change and radiographic progression in patients with rheumatoid arthritis in remission: A 5 year follow-up study. Rheumatology.

[15] Bouman CAM, van der Maas A, van Herwaarden N, Sasso EH, van den Hoogen FHJ, den Broeder AA (2017). A multi-biomarker score measuring disease activity in rheumatoid arthritis patients tapering adalimumab or etanercept: Predictive value for clinical and radiographic outcomes. Rheumatology.

[16] Ghiti Moghadam M, Lamers-Karnebeek FBG, Vonkeman HE, Ten Klooster PM, Tekstra J, Schilder AM (2018). Multi-biomarker disease activity score as a predictor of disease relapse in patients with rheumatoid arthritis stopping TNF inhibitor treatment. PLoS ONE.

[17] van der Heijde DM, van’t Hof MA, van Riel PL, Theunisse LA, Lubberts EW, van Leeuwen MA (1990). Judging disease activity in clinical practice in rheumatoid arthritis: First step in the development of a disease activity score. Ann. Rheum. Dis..

