# Supplementary material for: Relevance of circulating Semaphorin 4A for rheumatoid arthritis response to treatment
Source: Sci Rep. 2023 Sep 5;13:14626. doi: 10.1038/s41598-023-41943-3 (PMC10480203; doi:10.1038/s41598-023-41943-3)

Supporting information

**Relevance of circulating Semaphorin 4A for rheumatoid response to treatment**

**Jérôme Avouac (1,2,3), Eloïse Vandebeuque (1,2,3), Alice Combier (2,3), Lucile Poiroux (2,3), Alexia Steelandt (2,3), Margaux Boisson (2,3), Virginie Gonzalez (1,2), Anne Cauvet (1,2), Thomas Barnetche (4), Marie-Elise Truchetet (4), Chritophe Richez (4),Yannick Allanore (1,2,3)**

(1) INSERM U1016 and CNRS UMR8104, Institut Cochin, Paris, France

(2) Université de Paris, Université Paris Descartes, Paris, France

(3) Service de Rhumatologie, Hôpital Cochin, AP-HP.Centre - Université Paris Cité, Paris, France

(4) Service de Rhumatologie, FHU ACRONIM, Hôpital Universitaire de Bordeaux.

**Table S1: Targeted therapy modifications during the follow-up period because of insufficient disease control in cohort 1 from Paris**

| Patients | Discontinued Treatment | New treatment |
|---|---|---|
| P1 | Tocilizumab | Baricitinib |
| P2 | Tocilizumab | Rituximab |
| P3 | Etanercept | Baricitinib |
| P4 | - | Baricitinib |
| P5 | - | Etanercept |
| P6 | - | Rituximab |
| P7 | Abatacept | Baricitinib |
| P8 | Tocilizumab | Baricitinib |
| P9 | - | Etanercept |
| P10 | Infliximab | Abatacept |
| P11 | - | Adalimumab |
| P12 | Tocilizumab | Baricitinib |
| P13 | Etanercept | Tocilizumab |
| P14 | Infliximab | Abatacept |
| P15 | Rituximab | Baricitinib |
| P16 | Rituximab | Upadacitinib |
| P17 | Rituximab | Tocilizumab |
| P18 | - | Tocilizumab |
| P19 | - | Adalimumab |
| P20 | - | Baricitinib |
| P21 | Rituximab | Sarilumab |
| P22 | - | Abatacept |
| P23 | Adalimumab | Upadacitinib |
| P24 | Abatacept | Upadacitinib |
| P25 | - | Etanercept |
| P26 | Abatacept | Rituximab |

**Table S2: Predictive value of clinical, biological, and imaging variables for the occurrence of treatment failure (primary endpoint) and RA flares (secondary endpoint) in cohort 1 from Paris**

| Variable at baseline | Patients with DAS28-CRP <3.2 | |
|---|---|---|
| | **Treatment failure: RA flares AND Treatment escalation (HR, 95% CI)** | **RA flares (HR, 95% CI)** |
| SEMA4A >94 ng/mL | 3.50 (1.02-12.01) | 3.68 (1.33-10.17) |
| Synovial hyperemia on PDUS | 3.48 (0.75-16.21) | 2.79 (0.78-10.06) |
| Age | 1.00 (0.96-1.05) | 0.98 (0.94-1.02) |
| Active Smokers | 3.32 (0.60-7.21) | 2.67 (0.87-6.28) |
| Disease duration | 1.02 (0.97-1.08) | 0.99 (0.94-1.03) |
| Positive rheumatoid factor | 3.36 (0.43-26.30) | 5.26 (0.69-39.91) |
| Positive anti-CCP2 antibodies | 2.88 (0.37-22.59) | 4.41 (0.58-33.44) |
| Erosions on hand/foot x-rays | 2.77 (0.59-12.85) | 1.38 (0.48-3.99) |
| CRP levels >10 mg/L | 6.66 (0.77-57.62) | 3.80 (0.48-30.24) |
| Current treatment with corticosteroids | 0.77 (0.20-2.97) | 0.64 (0.22-1.88) |
| Line of Targeted therapy (>1 line vs. first line) | 3.15 (0.28-5.34) | 2.03 (0.31-4.98) |

*RA: rheumatoid arthritis, CRP: C-reactive protein, HR: Hazard Ratio; CI: Confidence Interval*

## Table S3: Baseline Characteristics of patients from the cohort 2 of Bordeaux

| | Patients with rheumatoid arthritis (n=40) | MTX initiators (n=15) | Tocilizumab initiators (n=25) |
|---|---|---|---|
| **Demographics** | | | |
| Age (years), mean ± SD | 57±14 | 51±15 | 60±13 |
| Females, n (%) | 29 (73) | 11 (73) | 18 (72) |
| **Disease characteristics** | | | |
| Disease duration (years), mean ± SD | 5±6 | 1±3 | 7±7 |
| Positive rheumatoid factor, n (%) | 27/34 (79) | 12/14 (86) | 15/20 (75) |
| Positive anti-CCP2 antibodies, n (%) | 28/34 (82) | 12/14 (86) | 16/20 (80) |
| Erosions on hand/foot x-rays, n (%) | 16 (40) | 3 (20) | 13 (52) |
| **Disease activity:** | | | |
| DAS28, mean ± SD | 5.12±1.40 | 4.36±0.95 | 5.58±1.45 |
| DAS28 >3.2, n (%) | 34 (85) | 11 (73) | 23 (92) |
| ESR (mmH1), mean ± SD | 43±31 | 35±29 | 47±31 |
| ESR>28 mmH1, n (%) | 24 (60) | 8 (53) | 16 (80) |
| CRP (mg/L), mean ± SD | 16±21 | 10±12 | 19±25 |
| CRP >10 mg/L, n (%) | 15/37 (41) | 4/14 (29) | 11/23 (48) |
| **Treatment received** | | | |
| Current corticosteroid use, n (%) | 26 (65) | 8 (53) | 18 (72) |
| Current corticosteroid use, >10 mg/day, n (%) | 0 (0) | 0 (0) | 0 (0) |
| Current conventional DMARD use, n (%) | 20 (50) | 0 (0) | 20 (80) |
| Current MTX use, n (%) | 18 (45) | 0 (0) | 18 (72) |
| History of targeted biologic therapies, n (%) | 10 (25) | 0 (0) | 10 (40) |

*DAS: Disease Activity Score, CRP: C-reactive protein, ESR: Erythrocyte Sedimentation Rate, HAQ: Health Assessment Questionnaire, MTX: Methotrexate, TNF: Tumor Necrosis Factor*

**Figure S1: Risk matrix assessing the predictive values of circulating SEMA4A in the cohort 1 from Paris, the DAS28 and the presence of synovial hyperemia on Power Doppler Ultrasounds (PDUS) for the further occurrence of treatment failure (primary endpoint) (A) and RA flares (secondary endpoint) (B)**

| | | | | | |
|---|---|---|---|---|---|
| **DAS28** | **>3.2** | 18% (4/22) | 53% (8/15) | + | **Active synovitis (PDUS)** |
| | | 20% (1/5) | 0% | - | |
| | **<3.2** | 29% (9/31) | 40% (2/5) | + | |
| | | 5% (1/20) | 33% (1/3) | - | |
| | | **≤94 ng/mL** | **>94 ng/mL** | | |
| | | **SEMA4A** | | | |

| | | | | | |
|---|---|---|---|---|---|
| **DAS28** | **>3.2** | 45% (10/22) | 73% (11/15) | + | **Active synovitis (PDUS)** |
| | | 40% (2/5) | 0% | - | |
| | **<3.2** | 38% (10/31) | 60% (3/5) | + | |
| | | 5% (1/20) | 33% (1/3) | - | |
| | | **≤94 ng/mL** | **>94 ng/mL** | | |
| | | **SEMA4A** | | | |

**Figure S2: Analysis of SEMA4A concentrations in the cohort 2 from Bordeaux**
A, Baseline circulating SEMA4A levels according to the Disease Activity Score (DAS) 28. Data are shown as the mean ± SEM. * p<0.05 determined by Student's t test. B, Baseline circulating SEMA4A levels according to therapeutic response assessed at month 3. Data are shown as the mean ± SEM. * p<0.05 determined by Student's t test. C-E, Course of SEMA4A concentrations according to response to therapy in the whole cohort (C), in methotrexate initiators (D) and tocilizumab initiators (E). * p<0.05, *** p<0.001 and ****p<0.0001, determined by the Wilcoxon matched-pairs signed rank test.

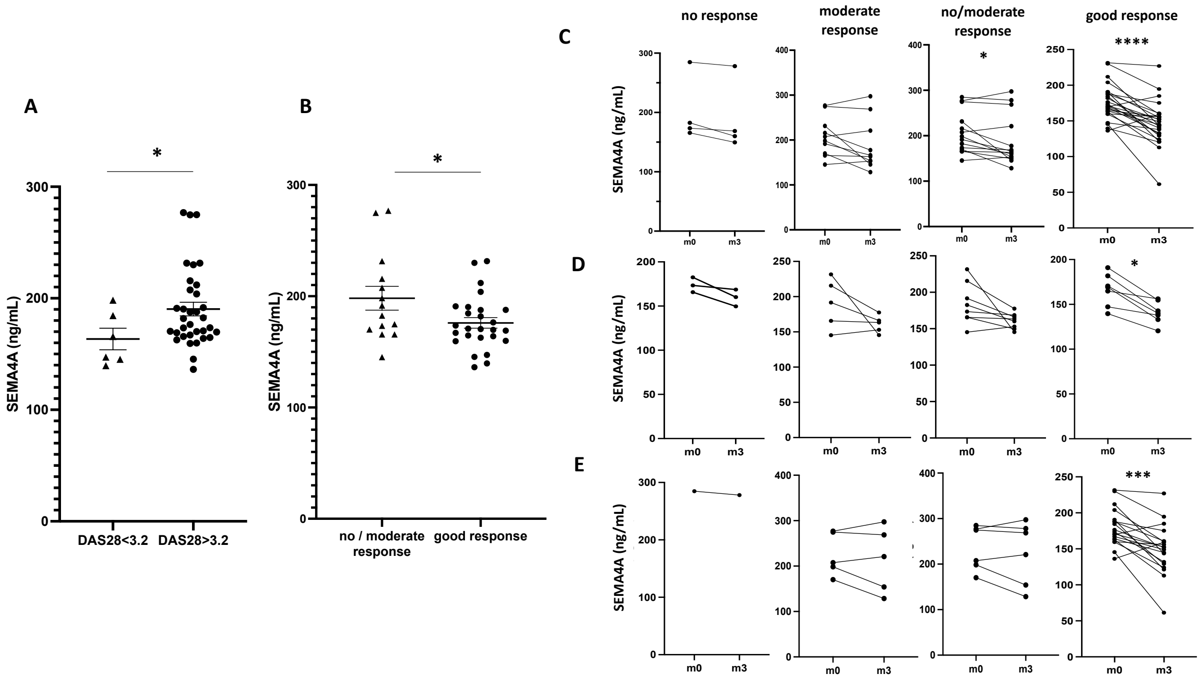

Supplement: Supplementary file 1 — Supplementary Information. [file 41598_2023_41943_MOESM1_ESM.pdf]
